# Clonal diversity of *Staphylococcus aureus* isolates in clinical specimens from selected health facilities in Ethiopia

**DOI:** 10.1186/s12879-023-08380-z

**Published:** 2023-06-12

**Authors:** Rajiha Abubeker. Ibrahim, Zelalem Mekuria, Shu-Hua Wang, Jose R. Mediavilla, Barry Kreiswirth, Eyasu T. Seyoum, Solomon H. Mariam, Wondwossen A. Gebreyes, Tesfa Addis Kefale, Geremew Tasew Guma, Nega Berhe

**Affiliations:** 1 Akililu Lemma Institute of Pathobiology, Addis Ababa University, Ababa, Ethiopia; 2 Ethiopian Public Health Institute, Addis Ababa, Ethiopia; 3 Ohio State Global One Health (GOH) LLC, Addis Ababa, Ethiopia; 4 Colleges of Veterinary Medicine, The Ohio State University, Columbus, OH USA; 5 Global One Health initiative (GOHi), The Ohio State University, Columbus, OH USA; 6 Infectious Disease Division, Internal Medicine Department, College of Medicine, The Ohio State University, Columbus, OH USA; 7 Center for Discovery and Innovation, Hackensack Meridian Health, Nutley, NJ USA

**Keywords:** Molecular epidemiology, *S. aureus*, Clinical specimen, Ethiopia

## Abstract

*Staphylococcus aureus* is among the top three causative agents of nosocomial infection in Ethiopia. The majority of studies in Ethiopia have focused on the epidemiology of *S. aureus* in hospital settings, with limited molecular genotyping results. Molecular characterization of *S. aureus* is essential for identification of strains, and contributes to the control and prevention of *S. aureus* infection. The aim of the current study was to determine the molecular epidemiology of methicillin-susceptible *S. aureus* (MSSA) and methicillin-resistant *S. aureus* (MRSA) isolates recovered from clinical specimens in Ethiopia. A total of 161 MSSA and 9 MRSA isolates were characterized using pulsed-field gel electrophoresis (PFGE) and staphylococcal protein A (*spa*) typing. Based on the PFGE analysis, MSSA isolates were grouped into eight pulso-types groups (from A to I), while MRSA isolates clustered into three (A, B and C) pulso-types with more than 80% similarity. The *spa* typing analysis showed diversity of *S. aureus* with 56 distinct *spa* types. *Spa* type t355 was most prevalent (56/170, 32.9%), while eleven new *spa* types were detected including t20038, t20039, and t20042. The identified *spa* types were clustered into 15 *spa*-clonal complexes (*spa*-CCs) using BURP analysis; novel/unknown *spa* types were further subjected to MLST analysis. The majority of isolates belonged to *spa*-CC 152 (62/170, 36.4%), followed by *spa*-CC 121 (19/170, 11.2%), and *spa*-CC 005 (18 /170, 10.6%). Of the nine MRSA isolates, 2 (22.2%) were *spa*-CC 239 with staphylococcal cassette chromosome (SCC)*mec* III. These findings highlight the diversity of *S. aureus* strains in Ethiopia, as well as the presence of potentially epidemic strains circulating in the country necessitating further characterization of *S. aureus* for antimicrobial resistance detection and infection prevention purposes.

## Background

*Staphylococcus aureus* is an important human pathogen causing a variety of infections in both healthcare facilities and community settings [1]. *S. aureus* is among the top three causes of nosocomial infection in Ethiopia. It was found to be the leading cause of nosocomial infection, accounting for 26.2% and 35.6% of infections in two hospitals in Ethiopia [2, 3]. In another hospital, *S. aureus* was the third leading cause, accounting for 20.6% of hospital-acquired infection [4]. The majority of studies were focused on antimicrobial susceptibility testing, with limited information on molecular epidemiology in Ethiopia [5, 6]. There was a single study showing the epidemiology of *S. aureus* using strain typing [7]. *S. aureus* are constantly changing, with novel strains appearing in different geographical regions [8–10]. Molecular characteristics of MRSA can be diverse in different hospitals within the same country [11].

Pulsed-field gel electrophoresis (PFGE), Multi Locus Sequence Typing (MLST) and staphylococcal protein A (*spa*) typing have been used extensively to identify different *S. aureus* strain types. For methicillin-resistant *S. aureus* (MRSA), molecular typing of the staphylococcal cassette chromosome (SCC)*mec*, which harbors the gene encoding methicillin resistance, provides additional strain discrimination [12].

The most frequently reported global MRSA clonal complexes (CCs) include the following: CC1, CC5, CC8, CC22, CC30, CC45, CC59 CC80, and CC239. Many of these are distribute globally, while others are restricted to particular region [13]. The most widely distributed healthcare associated (HA)-MRSA clone include ST239-MRSA-III, ST22- MRSA-IV while the most common community associated (CA)- MRSA clones include ST8-MRSA-IV (USA300), ST80-IV-MRSA and ST30-IV-MRSA which has been reported in many countries around the world. On the other hand, clones such as ST59-MRSA-IV and ST93-MRSA-IV have displayed comparatively restricted geographical spread. The pandemic HA-MRSA clone, ST239/ST241-III-MRSA has been reported since 1980 and 1990 s from most parts of the world including Africa, Australia, Europe, Asia, North and South America [14–16].

Although, genotypic reports of *S. aureus* and MRSA in Africa are limited, some have highlighted the major clones circulating within the continent. Commonly reported CC circulating in Africa include CC5, CC7, CC21, CC30, CC121 and CC152 [7, 17, 18], as well as MRSA strains with different SCC*mec* types belonging to CC1, CC8, CC22, and CC88. Notably, the globally-distributed HA-MRSA strain ST239/ST241-MRSA-III has only been identified in Egypt, Ghana, Kenya, and South Africa [19].

Molecular genotyping of *S. aureus* prospectively in healthcare setting can determine prevalent strains, identify outbreaks and transmission routes of newer strains, and implement control and prevention of *S. aureus* spread within healthcare settings. The aim of the current retrospective study was to determine the molecular epidemiology of MSSA and MRSA from multiple antimicrobial resistance (AMR) surveillance sites in Ethiopia to evaluate for any potential clusters outbreak transmissions prospectively.

## Methods

### Study site description

Ethiopia has been implementing a laboratory-based AMR surveillance program since 2016 [20]. Currently, more than 10 sentinel sites are networked within the national AMR surveillance system. Among these sites, four have been actively participating in the surveillance program since the program was initiated. The four sites included in this study were: Tikur Anbessa Specialized Hospital (TASH), Addis Ababa; Amhara Public Health Institute - Dessie Branch (APHI), Dessie; Ayder University Hospital (AUH), Mekelle; and the Clinical Bacteriology and Mycology National Reference Laboratory at the Ethiopian Public Health Institute (EPHI), Addis Ababa.

### Sampling strategy

A total of 190 stored *S. aureus* isolates from the aforementioned AMR Surveillance sites were characterized in this study. The clinical specimens included wound/pus (n = 167), blood (n = 8), ear swabs (n = 6), and other body fluids including eye swabs (n = 9). The isolates were collected from 2016 to 2019 from their respective sites and transported to EPHI. The isolates used in the study were stocked using 20% glycerol and tryptic soya broth in a cryotube and stored at -80 freezer for further analysis. The isolates were tested at the respective AMR surveillance sites using classical microbiological methods. Specimens were cultured on sheep blood agar plate (BAP) and beta-hematolytic colonies with characteristics indicative of *S. aureus* were further sub-cultured on mannitol salt agar (MSA). Yellow colonies were then sub-cultured onto nutrient agar and isolates were identified as *S. aureus* based on catalase and coagulase positivity. The isolates were then shipped to The Ohio State University (OSU) for molecular characterization.

### Diagnostic testing

All of the isolates were tested for antimicrobial susceptibility, *spa* type, Panton-Valentine leucocidin (*lukF-PV*), toxic shock syndrome toxin (*tst*), and 5 staphylococcal enterotoxin genes (*sea, seb, sec, seh, sej*) as previously described [21]. In addition, the isolates were characterized using PFGE, *spa* typing and the MRSA isolates were tested by using *SCCmec* typing. A subset of isolates with novel *spa* type patterns were also subjected to MLST analysis. The study protocol was approved by the EPHI Institutional Review Board (IRB) (Unique identifier: “EPHI-IRB-029-2017”). Data analyses were anonymous, and all phases of the study did not identify patients in any way.

### Nucleic acid isolation

Genomic DNA was extracted using a commercially available kit (QIAamp DNA mini Kits, Germany), following the manufacturer’s protocol [22]. DNA extraction and PCR tests were done at the Infectious Disease Epidemiology Molecular Laboratory (IDEML) at OSU. The extracts were stored at -20 °C until further analysis. MLST and *spa* typing was performed at the Center for Discovery and Innovation, Hackensack Meridian Health, Nutley, New Jersey, USA.

### Staphylococcal Cassette chromosome (SCC)*mec* typing and SCC*mec*-IV sub-typing

Nine MRSA isolates that were confirmed with *mecA* detection were tested for (SCC*mec typing* using PCR. Identification of cassette chromosome recombinase (*ccr*) alleles and *mec* class was used to determine SCC*mec* type as previously described [23]. For PCR master mix, illustra™ PuReTaq™ Ready-To-Go™ PCR Beads (GE Healthcare Bio-Sciences, USA) were used in 25 µl reactions, and reference strains of known SCC*mec* types were used as controls throughout the PCR test.

### Staphylococcal protein A (*spa*) typing

*Spa* typing was performed using PCR followed by Sanger sequencing, as previously described [24]. Assignment of *spa* type Based Upon Repeat Pattern (BURP) analysis for determination of *spa* clonal complex (*spa*-CC) was performed using Ridom StaphType. The Ridom SpaServer was used to predict the multi-locus sequence types (STs) as described previously [25]. Phylogenetic trees were constructed using RAxML Tree, Geneious Version 2022.2, (https://www.geneious.com).

### Multi-locus sequence typing (MLST)

MLST was performed using the method described by Enright et al. [26] for 12 isolates with novel/unknown *spa* type patterns (11 MSSA and 1 MRSA). Allelic sequences for each gene were analyzed using Geneious software, and used to query the *S. aureus* database (https://pubmlst.org/saureus/) [27]. Clonal complexes (CC) were inferred for 6 of the strains using the BURST analysis software available on the PubMLST server; the remainder were singletons or novel sequence types unrelated to any other in the database.

### Pulsed Field Gel Electrophoresis (PFGE)

PFGE was performed for further characterization of both MSSA and MRSA isolates. The isolates were selected randomly, while considering the site and specimen type to identify clonal relatedness. DNA fingerprinting was performed by macro-restriction of chromosomal DNA using *Sma*I (New England Biolabs, Ipswich, MA, USA) and pulsed field gel electrophoresis (PFGE) as described previously [28]. The PulseNet “universal” standard strain *Salmonella enterica* serovar Braenderup H9812 was used as a reference marker. The chromosomal fragments were separated using a CHEF-DR®III Pulsed-Field Electrophoresis System (Bio-Rad Laboratories, Hercules, CA, USA). Gel images were analyzed using Bionumerics Gelcompar II version 6. 6. software (Applied Math inc., Belgium). Cluster analysis was performed using the unweighted pair group method with arithmetic averages (UPGMA). Similarity coefficients were determined using Bionumerics by calculating the Dice coefficient similarity index. A similarity coefficient of 80% was selected to define individual pulso-types.

## Results

### Detection of virulence factor genes

Among the 190 total isolates, 172 were confirmed *S. aureus*. The remaining 172 *S. aureus* isolates tested for the presence of *spa* gene yielded positive results, while the *mec*A gene was detected in 9 of the isolates. None of the tested isolates were positive for *mecC*. Among the *S. aureus* isolates, 102 (59.3%) possessed the *lukF-PV* gene. A total of 66 (38.4%) isolates harbored at least one staphylococcal enterotoxin gene, while 31 (47.0%) isolates had more than one.

### Methicillin-susceptible *Staphylococcus aureus* (MSSA)

#### *Spa* typing

**Fig. 1:**
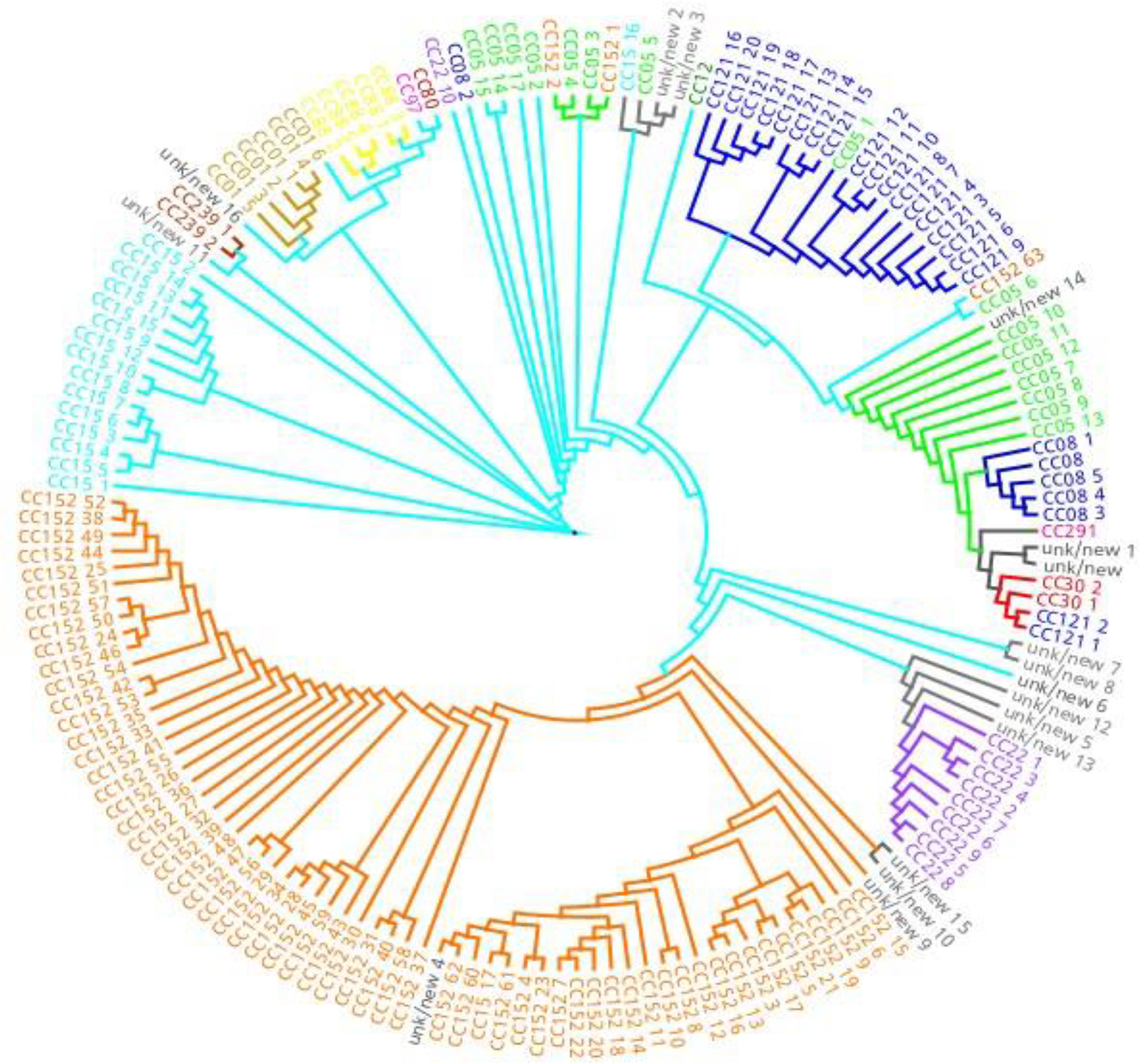
Clonal complexes of *Staphylococcus aureus* isolates identified by Based on Repeat Pattern BURP analysis. *spa*-CC, clonal complex

CC: Clonal complex; unk: unknown.

The *spa* typing analysis revealed 56 distinct *spa* types among the 170 *S. aureus* isolates, the most common being: t355 (56/170, 32.9%), t085 (13/170, 7.6%) and t314 (11/170, 6.5%). The other *spa* types had less than 5% frequency each. Eleven novel *spa* types were also identified (Table 1) and registered in the Ridom SpaServer database (https://spaserver.ridom.de/). The *spa* types identified were clustered into 15 *spa*-clonal complexes (*spa*-CCs) by BURP analysis; however, 18 of the isolates could not be identified by this method (Fig. 1). The majority of the isolates belonged to *spa*-CC 152 (62/170, 36.5%), and consisted of four *spa* types: t1172 [1], t1299 [3], t355(56) and t454[2]; followed by *spa*-CC 121 (19/170, 11.18%), *spa*-CC 5 (18 /170, 10.59%), *spa*-CC 15 (15/170, 8.82%), and *spa*-CC 22 (10 /170, 5.88%). MLST was performed (Table 2) for several *spa*-types which could not be assigned to *spa*-CC, including t777, t1916, t1991, and t5338.

**Table 1:** Novel *spa* types identified

Isolate #	location	*spa* type	*spa* repeat pattern	*spa*-CC
112	APHI	t20492	I2Z2EGMMMJH2G	CC 121
075	EPHI	t20034	ZEFGMDDMGMM	CC 101
072	EPHI	t20035	XBQBBMMM	CC 291
021*	EPHI	t20036	U-[r149]-O-[r149]	unknown
03*	EPHI	t20037	I2LI4KKQQQQQQ	unknown
052	EPHI	t20038	ZOKJBMMM	unknown
059*	EPHI	t20038	ZOKJBMMM	unknown
122	AUH	t20039	[r361]-DL-[r362]-M	unknown
128	AUH	t20039	[r361]-DL-[r362]-M	unknown
063*	EPHI	t20040	WBKKFFFMJI4	unknown
005*	EPHI	t20041	I2LOMLLML	unknown
093	APHI	t20042	[r361]-DL-[r362]-MMM	unknown
173	EPHI	t20042	[r361]-DL-[r362]-MMM	unknown
104	APHI	t20043	TJEJCMOMOK	CC 022

EPHI: Ethiopian Public Health institute; AUH: Ayder University Hospital; APHI: Amhara Public Health Institute. *spa*-CC, clonal complex; Isolates marked with an asterisk (*) were also subjected to MLST typing

### Multi-locus sequence typing (MLST)

Table 2 shows the MLST housekeeping genes, *spa* and clonal complex (CC) information for the 11 MSSA and one MRSA isolate. Among the 11 MSSA strains, 5 were assigned to CCs, including CC5, CC15, CC25, CC121 and CC398; these strains corresponded to *spa*-type t777, t605, t1916, t1991, and t5338, respectively. The remaining 6 MSSA strains displayed considerable variety, with multiple novel/unknown alleles in all 7 of the MLST housekeeping genes, which did not correlate to any existing sequence types (ST), nor to any known CCs. The isolate with *spa*-type t20037 is a double-locus variant of ST-5695, while isolate with *spa*-type t20038 is a single-locus variant of ST-2294; isolates with *spa*-types t20040 and t20041 are both singletons most closely related to ST-1852 and ST-5696, respectively; while isolate with *spa*-type t20036 is distantly related to ST-6984 (only matching 3 out of 7 loci). the closest match to the MRSA strain was ST 140 and was assigned to CC398; this corresponded to *spa*-types t3487.

**Table 2:** Multi-locus sequence typing housekeeping genes, *spa* type, and clonal complexes of methicillin-susceptible *Staphylococcus aureus* and methicillin-resistant Staphylococcus aureus isolates

ID	MSSA/MRSA	Ridom	7 MLST housekeeping genes							CC
			arcC	aroE	glpF	gmk	pta	tpiA	yqiL	
29	MSSA	t777	1	4	1	4	-	1	10	5
164	MSSA	t605	189	-	1	1	-	11	13	15
13	MSSA	t1916	4	1	4	1	-	5	4	25
123	MSSA	t1991	6	-	6	2	-	14	5	121
51	MSSA	t5338	3	35	48	19	805	26	39	398
40	MSSA	t6218	354	-	358	66	-	302	328	-
3	MSSA	t20037	354	-	-	169	-	-	-	-
5	MSSA	t20041	668	-	236	66	-	219	-	-
21	MSSA	t20036	-	-	-	66	-	-	-	-
63	MSSA	t20040	1	-	1	15	-	38	185	-
59	MSSA	t20038	-	-	45	-	-	153	5	-
147	MRSA	t3487	43	-	48	19	-	26	39	398

ID: sample identifier; MSSA: Methicillin susceptible *Staphylococcus aureus*; MRSA: Methicillin resistant *Staphylococcus aureus;* MLST: Multi-locus Sequence type; CC: Clonal complex;

### PFGE

Based on the PFGE analysis, MSSA isolates (n = 41) exhibiting more than 80% similarity were grouped into eight pulso-types (A, B, C, D, E, F, G, H and I) (Table 2). PFGE pulso-type D comprised 8 isolates (8/51 15.7%). Except for one isolate (body fluid), the pulso-type D clusters were all from pus specimens.

Pulso-types E and G consisted of 3 isolates each, whereas the other pulso-types comprised 2 isolates each. All the tested isolates have relatedness. The three isolates contained in a cluster and sub-clusters E, F and I were identified from sample all collected from EPHI. Cluster B strains were identified from sample collected from TASH and EPHI. Cluster A and C strains were identified from sample collected from TASH and APHI, and Dessie respectively. Cluster G strains were identified from sample collected from EPHI and AUH. Cluster H strains were identified from sample collected from APHI and AUH, Dessie and Mekelle. The PFGE pulso-types and *spa* CC showed close correlation of strains (Table 3).

**Table 3:** Methicillin Susceptible *Staphylococcus aureus* isolates pulsed field gel electrophoresis results using 80% similarity cut off

ID	site	specimen	Pulso-types	*spa* type	*spa*-CC
93	APHI	pus	NA	t20042	unknown
70	EPHI	ear swab	NA	t223	CC 22
47	TASH	pus	NA	t690	CC 88
54	TASH	pus	A	t85	CC 15
101	APHI	blood	A	t85	CC 15
42	EPHI	blood	NA	t85	CC 15
98	APHI	pus	NA	t84	CC 15
164*	TASH	pus	NA	t605	unknown
104	APHI	pus	NA	t20043	CC 22
163	TASH	pus	B	t2	CC 5
167	EPHI	body fluid	B	t355	CC 152
79	EPHI	pus	NA	t314	CC 121
112	APHI	pus	C	t20492	CC 121
148	TASH	pus	C	t355	CC 152
55	EPHI	pus	D	t355	CC 152
58	TASH	pus	D	t355	CC 152
95	APHI	pus	D	t355	CC 152
100	APHI	body fluid	D	t355	CC 152
121	AUH	pus	D	t355	CC 152
135	AUH	pus	D	t355	CC 152
142	APHI	pus	D	t355	CC 152
146	AUH	pus	D	t355	CC 152
71	EPHI	pus	E	t355	CC 152
73	EPHI	pus	E	t355	CC 152
87	EPHI	body fluid	E	t355	CC 152
80	EPHI	pus	F	t127	CC 1
88	EPHI	pus	F	t701	CC 8
74	EPHI	blood	NA	t213	CC 12
75	EPHI	body fluid	NA	t20034	CC 101
84	EPHI	ear swab	NA	t306	CC 5
89	EPHI	blood	NA	t17831	unknown
90	EPHI	pus	G	t306	CC 5
117	AUH	pus	G	t62	CC 5
156	AUH	pus	G	t314	CC 121
150	APHI	blood	H	t85	CC 15
114	AUH	pus	H	t127	CC 1
110	EPHI	pus	NA	t318	CC 30
46	EPHI	ear swab	I	t5084	CC 22
53	EPHI	pus	I	t223	CC 22
43	EPHI	body fluid	NA	t223	CC 22
40*	TASH	pus	NA	t6218	unknown

ID, isolate identification number; site, site of specimen collection; specimen, specimen type; *spa*-CC, clonal complex; NA, not applicable since the cut off value is set to 80% below that was not assigned a number; EPHI, Ethiopian Public Health institute; AUH, Ayder University Hospital; APHI, Amhara Public Health Institute; TASH, Tikur Anbessa Specialized Hospital; Isolate marked with an asterisk (*) was also subjected to MLST typing

### Methicillin Resistance *Staphylococcus aureus* (MRSA)

#### *Spa* typing

The 9 MRSA isolates were assigned to four *spa* clonal complexes (CC 8, CC 55, CC 88, and CC 239) and seven *spa* types (t30, t86, t306, t311, t688, t1476, t3487) as shown in (Table 4). One MRSA isolate (t3487) could not be characterized by BURP analysis; MLST analysis determined it as a single-locus variant of ST-140, previously associated with CC398.

**Table 4:** Staphylococcal Cassette Chromosome (SCC)*mec* and *spa* type of methicillin-resistant *Staphylococcus aureus* (MRSA) isolates

ID	location	SCC*mec* type	SCC*mec* IV sub-type	*spa-*type	*spa*-CC	Pulso types	Antimicrobial resistance	gene
18	EPHI	V	-	t311	CC 5	NA	PEN, OXA, CIP, LVX, ERY, ICLI, SXT	*seb*
28	AUH	IV	IVc	t306	CC 5	B	PEN, OXA, TET	*sea, seb, tsst*
111	AUH	VI	-	t688	CC 5	B	PEN, OXA, TET, SXT	*seb*
129	AUH	V	-	t1476	CC 8	NA	PEN, OXA, CIP, LVX, CLI, TET, RIF	*tsst*
131	EPHI	IV	IVa	t086	CC 88	A	PEN, OXA, TET, SXT	-
133	EPHI	IV	IVa	t086	CC 88	A	PEN, OXA, TET, SXT	-
24	EPHI	III	-	t030	CC 239	C	PEN, OXA, GEN, CIP, LVX, ERY, CLI, TET, NIT, RIF	*seb*
56	AUH	III	-	t030	CC 239	C	PEN, OXA, GEN, CIP, LVX, ERY, TET, NIT, RIF	*sea*
147*	AUH	IV	NT	t3487	unknown	NA	PEN, OXA, ICLI, ERY, CLI,	*seb, tsst*

AMR, Antimicrobial resistance; CIP, Ciprofloxacin; CLI, Clindamycin; ERY Erythromycin; GEN, Gentamicin; LVX, Levofloxacin; NIT, Nitrofurantoin; OXA, Oxacillin; PEN, penicillin; RIF, Rifampicin; TET, Tetracycline. EPHI: Ethiopian Public Health institute; AUH: Ayder University Hospital; APHI: Amhara Public Health Institute; NT non typable Isolate marked with an asterisk (*) was also subjected to MLST typing

### PFGE

**Fig. 2:**
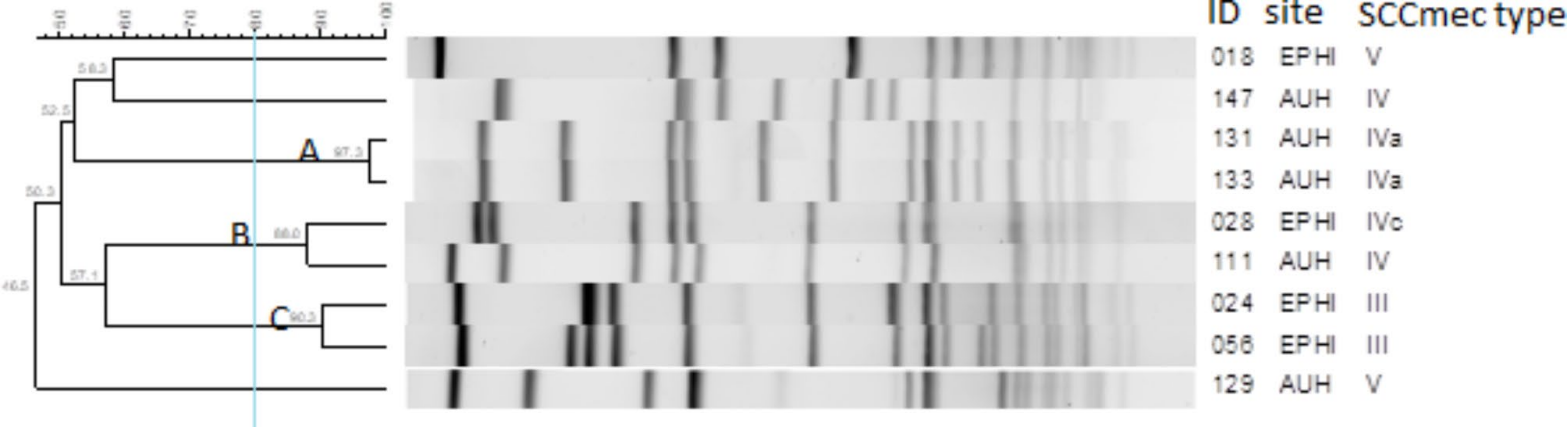
Pulsed-field gel electrophoresis dendrogram showing relatedness of methicillin resistant *Staphylococcus aureus* isolates

The PFGE analysis for the MRSA isolates (n = 9) yielded three pulso-types (A, B and C) with 93.7, 88.0, and 90.3% similarity, respectively. Each pulso-type consisted of two MRSA isolates (Fig. 2). Two isolates had 58.3% relatedness and the distant MRSA were 50.03% similar with other strains. All MRSA strains showed over 46% relatedness. The two isolates in pulso-type A had identical antimicrobial resistance patterns. Isolates in pulso-types B and C had similar AMR patterns Table 4.

### SCC*mec* typing and SCC*mec* IV sub-typing

Four of the nine (44.4%) MRSA isolates were found to be SCC*mec* type IV, 2/9 (22.2%) isolates were SCC*mec* type III, 2/9 (22.2%) isolates were SCC*mec* type V and 1/9 (11.1%) isolate was SCC*mec* type VI (Table 4).

## Discussion

The molecular epidemiology of *S. aureus* isolates circulating in Ethiopia has not been well-described previously. In this study, 56 different *spa* types and 15 *spa* clonal complexes were identified. Eleven of the *spa* types were novel, while six could not be assigned to *spa* or MLST clonal complexes, highlighting the diversity of *S. aureus* strains in Ethiopia. Among the *S. aureus spa* types identified, the most common included t085, t314 and t355. Interestingly, *spa*  type t355, clonal complex cluster CC152 is the most prevalent *spa* type reported in other east African countries [29]. This cluster was also the most prevalent in another study conducted in Ethiopia [7]. *Spa*-CC 152, *spa*-CC 5, *spa*-CC 8 and *spa*-CC 30 are among the most prevalent lineages identified in other African countries [17, 29, 30].

The strains characterized by MLST, were all associated with novel sequence types not found in the PubMLST database, with multiple unique alleles identified for each of the seven genes. Similar results were obtained in another study from Ethiopia, where more than half of the strains were shown to comprise novel STs with unique allelic combinations not found within the database [7]. The MRSA strain analyzed by MLST (*spa* type t3847) was shown to be closely related to ST-140, previously associated with CC398, a livestock-associated lineage [31]. This strain was reported on inanimate objects and from patient infection associated with hospital transmission elsewhere in Africa [18, 32].

Among the MSSA isolates, PFGE analysis identified eight distinct pulso-types, as well as several distantly-related strains. The PFGE results indicated that strains from different geographical areas were genotypically related. Another PFGE-based study conducted on *S. aureus* isolates from distinct regions of Ethiopia reported similar patterns [33]. The presence of similar strains in widespread areas is possibly related to the large-scale movement of people within Ethiopia, especially to the central region where the capital city Addis Ababa is located. In this study, the *spa* type assignments were closely correlated with PFGE pulso-types, similar to what has been described in other studies [34].

The three clusters of MRSA strains displayed relatedness regardless of geographic separation. One strain was found to exhibit > 80% relatedness with strains from AUH, Mekelle and EPHI. In addition, all MRSA isolates showed more than 46% similarity, suggesting relatedness between strains from different geographic location. Moreover, strains clustered within a single pulso-type also showed similar antimicrobial resistance properties. Other studies have also highlighted correlations between strain relatedness and antimicrobial resistance patterns [35, 36].

In this study, the most common *spa*-CC for MRSA strains were *spa-CC* 5, comprising SCC*mec* types, IV, V, and VI, and *spa* types t306, t311 and t688. Another study from Africa also reported *spa*-CC 5 as the most common lineage among MRSA strains [30, 37]. Previously, SCC*mec* types I–III were considered to be HA-MRSA, whereas SCC*mec* types IV and V were considered CA-MRSA [38]. In recent years, however, the distinction between HA-MRSA and CA-MRSA has blurred increasingly in recent years, as a growing number of reports have demonstrated that CA-MRSA lineages are now prevalent in hospitals [39, 40].

Two MRSA strains displayed 97.3% similarity using PFGE, and had identical SCC*mec* (type IVa) and *spa* (t086) types, as well as identical antimicrobial resistance patterns. Another two of MRSA strains were *spa*-CC 239 with SCC*mec* type III, and displayed multidrug resistance, suggestive of HA-MRSA. This result was consistent with other studies describing CC 239 SCC*mec* type III as being commonly associated with multidrug resistance and treatment failure [41, 42]. Moreover, CC 239 SCC*mec* type III was known to cause MRSA pandemic, circulating in many countries and also associated with serious illness such as admittance to ICU and high rate of death [16, 43].

Despite identification of some cluster, the study is limited by the retrospective nature of the isolates tested and lack of clinical and epidemiological information for linking the cases. In addition, the use of PFGE and spa typing are primarily used for local epidemiological investigation and cannot be applied to multiple sites without knowing if these are the endemic strains versus emerging outbreak strains. Unfortunately, more robust genotyping with MLST for all the isolates and/or whole genome sequence (WGS) could not be performed due to budgetary constraints.

## Conclusion

The most predominant *spa* type in Ethiopia was found to be t355, belonging to *spa*-CC 152. The *spa* types identified in this study were closely associated with the PFGE pulso-types. Eleven new *spa* types were identified among the MSSA isolates, while among the MRSA isolates, strains with high antimicrobial resistance and global epidemic potential were identified. These findings highlight the diversity of *S. aureus* strains in Ethiopia, as well as the presence of potentially epidemic strains circulating in the country necessitating further characterization of *S. aureus* for antimicrobial resistance detection and infection prevention purposes in prospective study with MLST and/or WGS for country wide analysis.

## Data Availability

The datasets used and/or analyzed during the current study are available from the corresponding author on reasonable request.
